# Psychopathological characteristics of patients eligible for a diacethylmorphine prescription program: an ecological pilot study

**DOI:** 10.1192/j.eurpsy.2022.744

**Published:** 2022-09-01

**Authors:** I. Gothuey, M. Jeannin, R. Bouzegaou, A. Kuntz, V. Salamin

**Affiliations:** 1 Fribourg University, Science And Medecine Faculty, Fribourg, Switzerland; 2 Freiburg Mental Health Network, Adult Psychiatric Department, Marsens, Switzerland; 3 Freiburg Mental Health Network, Adult Psychiatric Department, Fribourg, Switzerland

**Keywords:** agonist opiate treatment, diacethylmorphine treatment, Addiction, dual disorders

## Abstract

**Introduction:**

Agonist opiate treatments with diacethylmorphin (DAM) for heroin addiction have proven their effectiveness for a long time. But few studies focused on psychiatric troubles among the treated patients. As a new DAM program will open in Freiburg in Switzerland, in order to assess the eligibility to this program, we consider the psychiatric dimension using the Addition Severity Index French translation (IGT).

**Objectives:**

Assessing the patient eligibility for the DAM programm and describing psychopathological characteristics

**Methods:**

Assessing eligibility for a Dam program in Switzerland is based on some criteria defined by OFSP: Be adult, failure of at least two previous addiction treatments, intravenous consumption. In addition, the included patients (N=10) passed an interview with a trained examiner, to fill the addiction severity index scale (multidimensional psychometric scale). The result of the psychiatric dimension of IGT was compared with the psychiatric diagnosis in the medical file to assess the internal reliability of the descriptive method. Statistical method for little sample, mean, median, descriptive datas and Fisher test were applied.

**Results:**

All kind of affective disorders, were the most representative psychiatric trouble in the studied population (47%) followed by personality disorders (32%) and severe anxiety troubles (21%). The psychiatric dimensional evaluation of IGT was consistent with the description file psychiatric diagnosis. In a surprising way, we found no psychosis spectrum troubles who could explained the previous treatment failure.

**Conclusions:**

Affective disorders are overrepresented in our sample of addicted patient included in the DAM program. These troubles stay often underestimated. The have to be properly treated

**Disclosure:**

No significant relationships.

